# Pan-cancer characterization of lncRNA modifiers of immune microenvironment reveals clinically distinct de novo tumor subtypes

**DOI:** 10.1038/s41525-021-00215-7

**Published:** 2021-06-17

**Authors:** Zicheng Zhang, Congcong Yan, Ke Li, Siqi Bao, Lei Li, Lu Chen, Jingting Zhao, Jie Sun, Meng Zhou

**Affiliations:** 1 School of Biomedical Engineering, School of Ophthalmology & Optometry and Eye Hospital, Wenzhou Medical University, Wenzhou, China; 2 Institute of Systems and Physical Biology, Shenzhen Bay Laboratory, Shenzhen, China

**Keywords:** Tumour biomarkers, Data mining

## Abstract

The emerging field of long noncoding RNA (lncRNA)-immunity has provided a new perspective on cancer immunity and immunotherapies. The lncRNA modifiers of infiltrating immune cells in the tumor immune microenvironment (TIME) and their impact on tumor behavior and disease prognosis remain largely uncharacterized. In the present study, a systems immunology framework integrating the noncoding transcriptome and immunogenomics profiles of 9549 tumor samples across 30 solid cancer types was used, and 36 lncRNAs were identified as modifier candidates underlying immune cell infiltration in the TIME at the pan-cancer level. These TIME lncRNA modifiers (TIL-lncRNAs) were able to subclassify various tumors into three de novo pan-cancer subtypes characterized by distinct immunological features, biological behaviors, and disease prognoses. Finally, a TIL-lncRNA-derived immune state index (TISI) that was reflective of immunological and oncogenic states but also predictive of patients’ prognosis was proposed. Furthermore, the TISI provided additional prognostic value for existing tumor immunological and molecular subtypes. By applying the TISI to tumors from different clinical immunotherapy cohorts, the TISI was found to be significantly negatively correlated with immune-checkpoint genes and to have the ability to predict the effectiveness of immunotherapy. In conclusion, the present study provided comprehensive resources and insights for future functional and mechanistic studies on lncRNA-mediated cancer immunity and highlighted the potential of the clinical application of lncRNA-based immunotherapeutic strategies in precision immunotherapy.

## Introduction

Cancer is a highly complicated and delicate disease influenced by not only genetic/epigenetic changes in the tumor cells but also the surrounding complex and dynamic milieu known as the tumor microenvironment (TME)^1,2^. The mutual and dynamic crosstalk among cellular and molecular components of the TME serves profound roles in tumor initiation, progression, and metastasis^3,4^. The immunological components of the TME have been recognized as essential hallmark features of the TME through the formation of a vital specialized microenvironment known as the tumor immune microenvironment (TIME)^5^. Recent advances in the precise dissection of the TIME have demonstrated that infiltrating immune cells exert multiple functions in the complex ecosystem of the TME, and their complexity and diversity within the TME can exert both pro- and antitumorigenic effects, as well as affect a variety of clinical outcomes and therapeutic responses, particularly the response to immunotherapy^6,7^.

Considerable evidence has suggested that the TIME is highly dynamic and plastic during tumor progression and therapeutic interventions, and is determined and remodeled by genetic alterations of oncogenic signaling, genetic and epigenetic regulators, and cellular metabolism^8–10^. Over the last few years, long noncoding RNAs (lncRNAs) have emerged as critical players in gene regulatory networks, affecting diverse biological and physiological processes^11,12^. Recent progress in functional studies has also highlighted the crucial roles of lncRNAs in the development and functions of the immune system, and their potential to regulate all aspects of immunity^13–15^. lncRNAs are expressed in various immune cell types, preferentially in a lineage-specific manner, and contribute to immune cell development, differentiation, and activation^16–18^. Increasing evidence also shows that lncRNAs can function as communicators and mediators, being directly and/or indirectly involved in the crosstalk between tumor cells and infiltrating immune cells within the TIME to participate in cancer onset and progression^19–24^. Furthermore, certain lncRNAs have been shown to influence and regulate the migration and infiltration of immune cells within the TIME, and are associated with tumor immune evasion and prognosis^24,25^. For example, Yang et al.^26^ identified lncRNA *EPIC1* as a regulator of tumor immune evasion and response by suppressing tumor cell antigen presentation. lncRNA *TCL6* was found to be correlated with immune cell infiltration and poor survival in breast cancer^27^.

Despite the evidence that suggests the involvement of lncRNAs in remodeling the TIME, the known TIL-lncRNAs of infiltrating immune cells are limited and need to be characterized further. In the present study, a systems immunology framework was used to identify potential lncRNA modifier candidates of infiltrating immune cells within the TIME through an integrative analysis of the noncoding transcriptome and immunogenomics profile of 9549 tumor samples across 30 solid cancer types. The specific impact of TIL-lncRNAs on tumor behavior and disease prognosis was also investigated herein.

## Results

### Derivation of de novo pan-cancer subtypes associated with distinct immunological features from the perspective of immune cell infiltration-lncRNA crosstalk

**Fig. 1:**
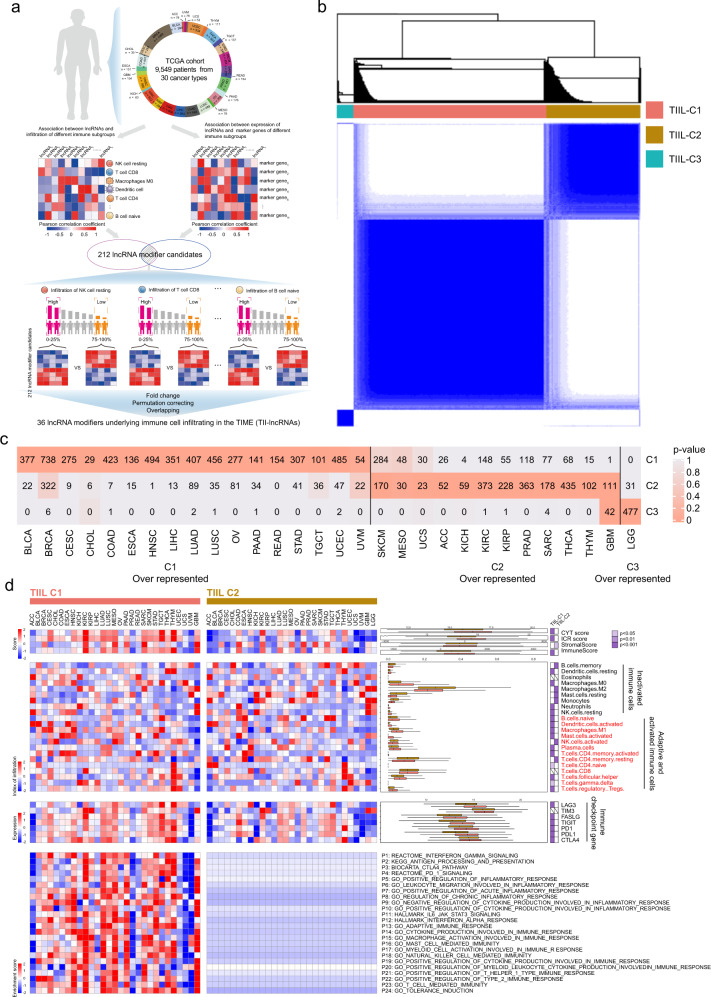
Derivation of de novo pan-cancer subtypes based on TII-lncRNAs. **a** Schematic illustration of the systems immunology framework for identifying potential lncRNA modifier candidates of infiltrating immune cells within the TIME. **b** Consensus clustering matrix of 9549 TCGA pan-cancer samples based on RNA-seq expression values of 36 TII-lncRNAs. **c** The number of cases in each cluster across tumor types. Colors in the boxes represent the *P*-value calculated from a hypergeometric test that compared the proportion of samples with a known cancer type in a cluster to the overall proportion of samples in that cluster. Red represents the enrichment level. **d** Comparison of immunological features within the TIME between de novo pan-cancer subtypes defined by TII-lncRNAs. lncRNA long noncoding RNAs, TII-lncRNAs TIME lncRNA modifiers, TCGA The Cancer Genome Atlas.

In the present study, a systems immunology framework was proposed to identify potential lncRNA modifier candidates of infiltrating immune cells within the TIME by considering the correlation between lncRNA expression and immune-related molecular and cellular components of the TIME at the pan-cancer level (Fig. 1a). A total of 36 lncRNAs were identified as modifier candidates underlying immune cell infiltrating in the TIME (referred to as TII-lncRNAs; Supplementary Table 1). To explore whether molecular subgroups could be discovered by these TII-lncRNAs, as opposed to pan-cancer subtypes previously identified using other transcriptomic features, consensus clustering was conducted for all TCGA patients based on the expression pattern of 36 TII-lncRNAs, which uncovered three major pan-cancer subtypes (referred to here as TIIL-C1 to -C3; Fig. 1b). The patient distribution of each cancer type across the three subtypes was examined, and the ES was calculated by comparing the number of patients with a given cancer type in each subtype with those of any cancer types in this subtype, and by performing the hypergeometric test to assess statistical significance, as reported in Xie’s study^28^. As shown in Fig. 1c, 17/30 cancer types were over-represented in the TIIL-C1 subtype and 12 cancer type were over-represented in the TIIL-C2 subtype. Brain tumors (GBM and brain lower grade glioma) were over-represented in the TIIL-C3 subtype. Consequently, the subsequent experiments focused on the TIIL-C1 and TIIL-C2 subtypes, where cancer types were relatively uniformly distributed.

Next, the characteristics of the TIME were examined by comparing infiltrating stromal and immune cell populations, immune effector activity, immune-mediated tissue-specific destruction, expression of immune-checkpoint genes (ICGs), and immune pathway activity between the TIIL-C1 and TIIL-C2 subtypes. As shown in Fig. 1d, the TIIL-C1 subtype showed significantly higher immune and lower stromal cell scores compared with the TIIL-C2 subtype, as determined by Wilcoxon rank-sum test (*P* < 0.001; Fig. 1d). The ICR and CYT scores were also significantly higher in the TIIL-C1 subtype compared with those in the TIIL-C2 subtype, as determined by the Wilcoxon rank-sum test (*P* < 0.001; Fig. 1d). Further detailed analysis of the relative abundance of 22 immune cell populations using the deconvolution method revealed that the TIIL-C1 subtype had significantly higher levels of adaptive and activated immune cell infiltration, whereas the TIIL-C2 subtype demonstrated a low infiltration of adaptive and activated immune cells and high infiltration of inactivated immune cells. Furthermore, ICGs tended to be significantly upregulated in the TIIL-C1 subtype compared to those in the TIIL-C2 subtype, as determined by Wilcoxon rank-sum test (*P* < 0.001; Fig. 1d). The pathway enrichment analysis by ssGSEA showed that the TIIL-C1 subtype could be characterized by upregulated immune pathways, as compared with the TIIL-C2 subtype (Fig. 1d). Overall, these results demonstrated that the TIIL-C1 subtype may be associated with an immune-active microenvironment phenotype and the TIIL-C2 subtype with an immune-silent microenvironment phenotype.

To confirm the stability and reliability of TII-lncRNA-derived subtype classification, randomized analysis was performed by randomly removing 40 or 60% of samples and conducting the same consensus clustering for the remaining 60 or 40% of samples based on the expression of 36 TII-lncRNAs (Fig. S1). The results of the randomized analysis were the same as those of the analysis of the entire TCGA pan-cancer samples, indicating the classification effectiveness and robustness with 36 TII-lncRNAs.

### Biological and clinical differences across TII-lncRNA-derived pan-cancer subtypes

**Fig. 2:**
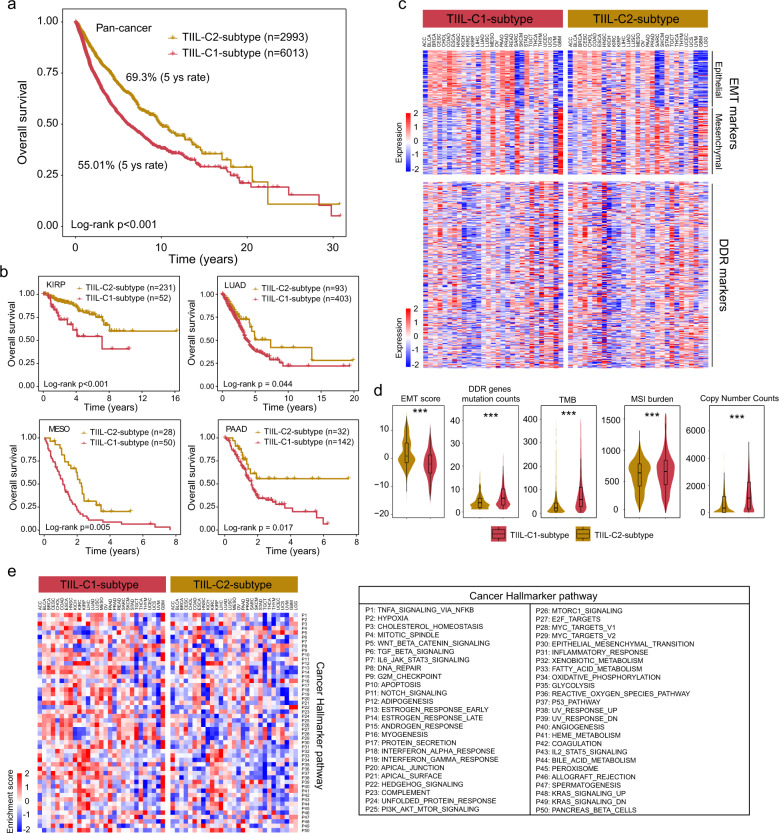
Biological and clinical heterogeneity across TII-lncRNA-derived pan-cancer subtypes. **a** Pan-cancer Kaplan–Meier survival curves of tumors in patients with the TIIL-C2 and TIIL-C1 subtypes. The *P*-value was calculated by the log-rank test. **b** Kaplan–Meier survival curves of tumor patients with KIRP, LUAD, MESO, and PAAD. The *P*-value was calculated by the log-rank test. **c** Heatmap of the expression of EMT and DDR markers between the TIIL-C1 and TIIL-C2 subtypes. **d** Boxplot for genomic alteration features between the TIIL-C2 and TIIL-C1 subtypes. The *P*-value was calculated by two-sided Wilcoxon rank-sum tests (***P < 0.001). **e** Heatmap of ssGSEA enrichment score of cancer hallmark pathways between the TIIL-C1- and TIIL-C2-subtype. lncRNA long noncoding RNAs, TII-lncRNAs TIME lncRNA modifiers, KIRP kidney renal papillary cell carcinoma, LUAD lung adenocarcinoma, MESO mesothelioma, PAAD pancreatic adenocarcinoma, EMT epithelial-mesenchymal transition, DDR DNA Damage Response, ssGSEA single-sample Gene Set Enrichment Analysis.

Next, the clinical relevance of different TII-lncRNA-derived subtypes was explored. Pan-cancer survival analysis revealed that the TII-lncRNA-derived subtypes were significantly associated with patient survival (HR = 1.872, 95% CI = 1.709–2.05, *P* < 0.001; Fig. 2a), and that the TIIL-C2 subtype had a significantly superior overall survival than the TIIL-C1-subtype (log-rank test *P* < 0.001; Fig. 2a). Cancer-specific survival analysis also revealed an association between the TII-lncRNA-derived subtypes and overall survival in multiple cancer types, with a similar prognostic trend as that observed in the pan-cancer analysis (Fig. 2b).

It was then further examined whether tumors in different TII-lncRNA-derived subtypes had different biological features, including EMT process, DNA damage response (DDR), and hallmark gene expression. By examining the EMT status of the tumors, it was found that EMT signature scores of tumors were significantly lower (and more epithelial) in the TIIL-C1 subtype than in the TIIL-C2 subtype. A total of 81/94 epithelial markers were upregulated in tumors from the TIIL-C1 subtype, whereas 74/111 mesenchymal markers were upregulated in the TIIL-C2 subtype. A total of 169/309 DDR genes were found to be upregulated in the TIIL-C1 subtype compared with the TIIL-C2 subtype (Fig. 2c). Furthermore, a significant difference in the overall number of DDR gene mutations was observed between the TIIL-C1 and TIIL-C2 subtypes, as determined by the Wilcoxon rank-sum test (*P* < 0.001; Fig. 2d). Clear links between genomic alteration features and the TIME have been reported. Therefore, genomic alteration features were further investigated in the two TII-lncRNA-derived pan-cancer subtypes. Overall, the TIIL-C1 subtype was characterized by a high level of tumor mutational burden, microsatellite instability, and copy number variation (Fig. 2d). Geneset enrichment analysis by ssGSEA showed that the TIIL-C1 subtype was associated with high activation of nearly all cancer hallmark pathways (Fig. 2e). In combination, these findings suggested that the biological and clinical behaviors of tumors in different TII-lncRNA-derived subtypes were heterogeneous.

### Development of a TISI

**Fig. 3:**
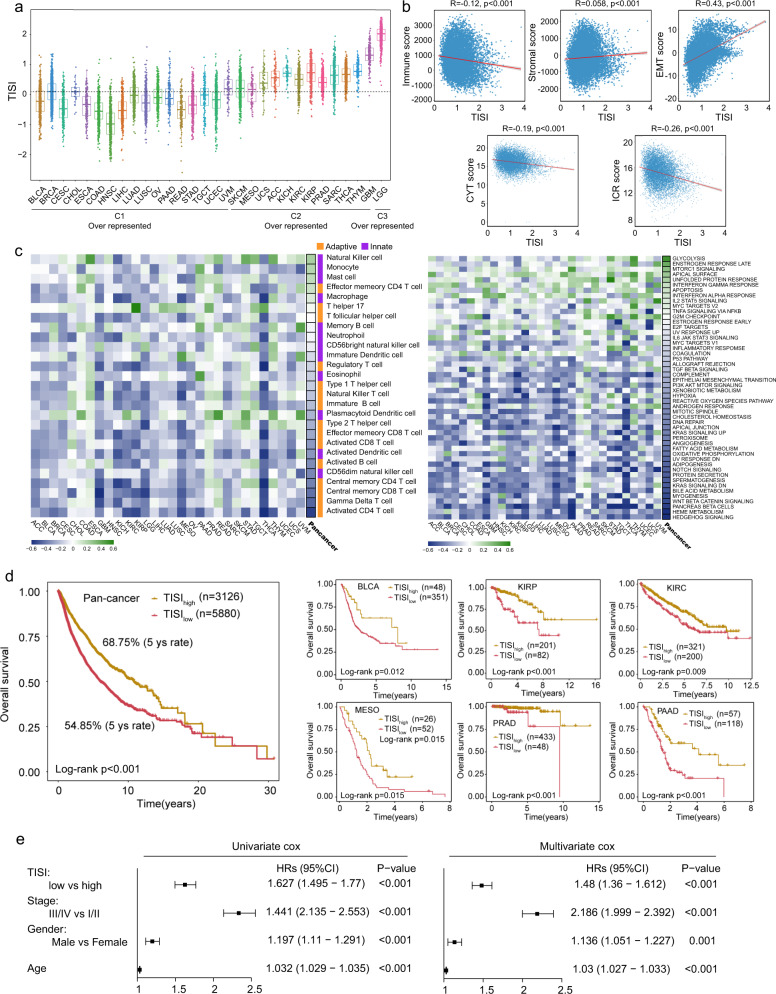
Development and validation of the TISI in pan-cancer. **a** Distribution of TISI in different cancer types. The dotted line represents the median TISI in all samples. **b** Correlation between cancer-related events and the TISI. **c** Association of the infiltration status of different immune cell populations and cancer hallmark pathways with the TISI. **d** Pan-cancer and cancer-specific Kaplan–Meier survival curves of tumor patients between TISI_high_ and TISI_low_ groups. The *P*-value was calculated by the log-rank test. **e** Univariate and multivariate analysis of the TISI with other standard clinical features in the pan-cancer. *P*-value was calculated by the Cox proportional hazard model. lncRNA long noncoding RNAs, TISI TIL-lncRNA-derived immune state index.

Considering the association of TII-lncRNA-derived subtypes with the TIME and biological and clinical behaviors, a TISI was constructed by calculating the mean expression levels of 36 TII-lncRNAs to reflect the affinity immune phenotype of a tumor. As shown in Fig. 3a, cancer types that were over-represented in the TIIL-C1-subtype exhibited a lower TISI, whereas those over-represented in the TIIL-C3-subtype exhibited a higher TISI. Furthermore, the TISI exhibited a significant negative correlation with the immune (R = −0.12, *P* < 0.001), CYT (R = −0.19, *P* < 0.001), and ICR (R = −0.26, *P* < 0.001) scores, and a significant positive correlation with the stromal (R = 0.058, *P* < 0.001) and EMT (R = 0.43, *P* < 0.001) scores (Fig. 3b).

Next, the association between the infiltration status of different immune cell populations and the TISI was examined, and the TISI was found to be negatively correlated with the abundance of adaptive immune cell populations, such as activated CD4 T cells, γδ T cells, central memory CD8 T cells, and central memory CD4 T cells, and positively correlated with the abundance of innate immune cell populations, such as natural killer cells, monocytes, and mast cells (Fig. 3c). This observation suggested that the TISI has great potential in capturing the antitumor immunity of tumors. Further hallmark pathway enrichment analysis also revealed the enrichment of pathways involved in immunosuppression, such as glycolysis, late estrogen response, and mTOR complex 1 signaling, which were found to be significantly positively correlated with the TISI, whereas the enrichment of most cancer-related pathways was found to be negatively correlated with the TISI. These results demonstrated that the TISI was not only associated with intertumoral immune states but also reflected oncogenic states, which were predictive of patient outcomes.

Therefore, the prognostic significance of the TISI in predicting pan-cancer survival was examined. Pan-cancer survival analysis revealed that the TISI was significantly associated with patient survival (HR = 1.627, 95% CI = 1.495–1.770, *P* < 0.001; Fig. 3d). As shown in Fig. 3d, patients with a high TISI had a significantly improved survival compared with those with a low TISI (log-rank *P* < 0.001). Cancer-specific survival analyses also revealed a significant association between the TISI and survival in multiple cancer types, with a similar prognostic trend observed in the pan-cancer analysis (Fig. 3d). Furthermore, the TISI maintained a significant association with survival after adjusting other standard clinical features in the pan-cancer analysis (Fig. 3e).

### The TISI provides additional prognostic value for tumor immunological and molecular subtypes

**Fig. 4:**
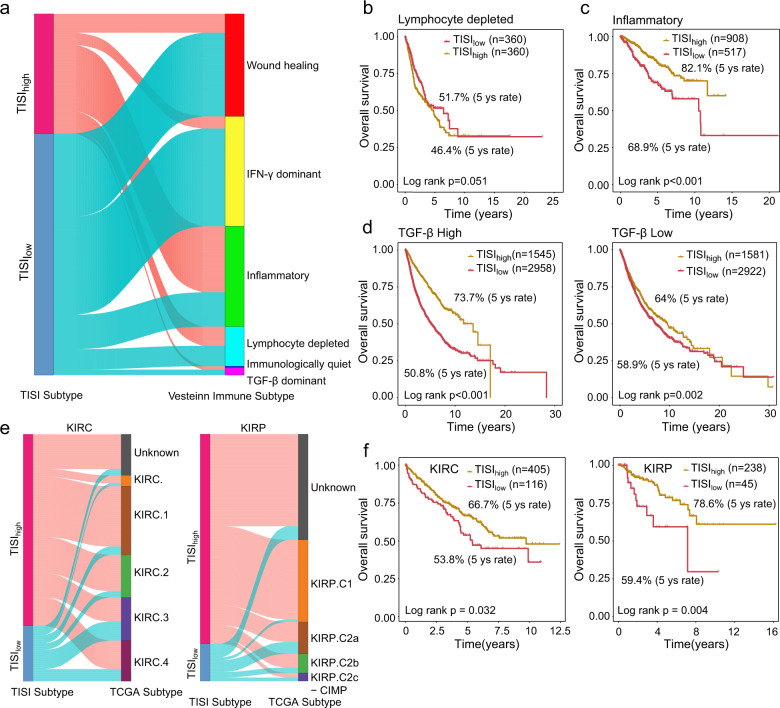
Concordance between TISI-defined risk groups and immunological and molecular subtypes. **a** Alluvial diagram showing the association between TISI-defined risk groups and immunological subtypes. **b**–**d** Kaplan–Meier survival curves of tumor patients between the TISI_high_ and TISI_low_ groups in several immunological subtypes. The *P*-value was calculated by the log-rank test. **e** Alluvial diagram showing the association between TISI-defined risk groups and molecular subtypes of KIRC and KIRP. **f** Kaplan–Meier survival curves of tumor patients in the TISI_high_ and TISI_low_ groups in KIRC and KIRP. The *P*-value was calculated by the log-rank test. TISI TIL-lncRNA-derived immune state index, KIRC kidney renal clear cell carcinoma, KIRP kidney renal papillary cell carcinoma.

The correlation between TISI-defined risk groups and immune subtypes (ISs) defined by immune signature sets was further investigated^29^. As shown in Fig. 4a, TISI-defined risk groups were present in all ISs, but their relative distribution differed among ISs. The C1-wound healing, C2-interferon (IFN)-γ dominant and C6-transforming growth factor-β (TGF-β) dominant ISs were particularly dominant in the low TISI group, whereas the high TISI group was enriched in the C3-inflammatory and C5-immunologically quiet ISs. A relatively equal distribution for TISI-defined risk groups was observed in C4-lymphocyte depleted ISs, which was consistent with the fact that C4-lymphocyte depleted ISs include subtypes with mixed signatures, whose prognosis is dependent on immune contexts (Fig. 4a). Further observation indicated that some of the low TISI tumors also occurred in the C3-inflammatory ISs, with some high TISI tumors occurring in the C6-TGF-β dominant ISs. Therefore, stratification analysis of the TISI for C3-inflammatory, C4-lymphocyte depleted, and C6-TGF-β dominant ISs was conducted. Although the C3-inflammatory IS has been reported to have the best prognosis, the TISI was still able to effectively identify a subgroup of patients at high risk for poor prognosis from the C3-inflammatory IS (log-rank *P* < 0.001; Fig. 4c). Although tumors in the C4-lymphocyte depleted and C6-TGF-β dominant ISs are known to have the least favorable outcome, the TISI revealed a good prognostic prediction ability and could effectively distinguish high- and low-risk patients for the same C4-lymphocyte depleted (log-rank *P* = 0.051) and C6-TGF-β dominant ISs (Fig. 4b and d). For patients with high and low TGF-β signaling, a high TISI was significantly associated with improved survival compared with low TISI (log-rank *P* < 0.001 for high TGF-β signaling, and *P* = 0.002 for low TGF-β signaling; Fig. 4d). The opposite trend was observed in the C4-lymphocyte depleted IS, in which high TISI was marginally significantly associated with poor survival than low TISI (Fig. 4b).

The interplay of TISI-defined risk groups with the previously defined mRNA-based consensus molecular subtypes (CMSs) was next assessed, and kidney renal clear cell carcinoma (KIRC) and kidney renal papillary cell carcinoma (KIRP) were selected as case studies. It was found that TISI-defined risk groups spanned across CMSs and had no substantial heterogeneity in the distribution of CMS subgroups (Fig. 4e). However, the TISI could effectively stratify patients in the different CMS subgroups of KIRC and KIRP into different risk groups with significantly different overall survival rates (Fig. 4f). As shown in Fig. 4f, patients with a high TISI exhibited a significantly improved survival compared with those with a low TISI (log-rank *P* = 0.032 for KIRC and log-rank *P* = 0.004 for KIRP).

### Potential of TISI as a predictor of immunotherapy response

**Fig. 5:**
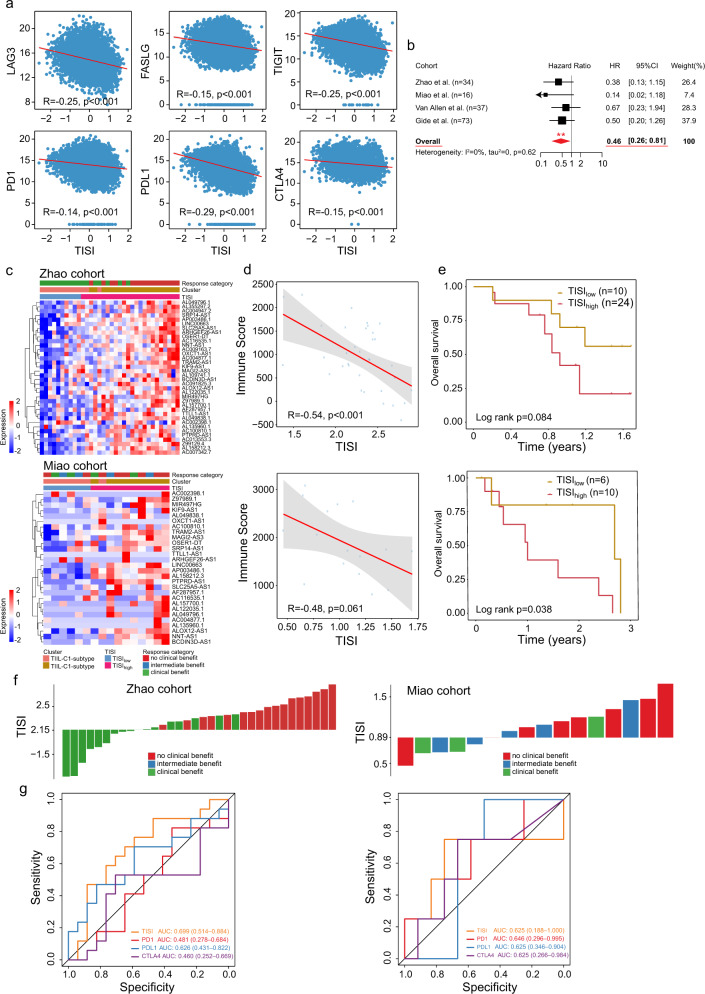
Association between the TISI and the clinical response to immunotherapy. **a** Association between the TISI and expression of immune-checkpoint genes. **b** Forest plot visualizing the HRs of univariate survival analysis of the TISI in four immunotherapy cohorts. Significance was determined using the Cox proportional hazard model. The red diamond shows the random-effects meta-analysis summary of HRs over four immunotherapy cohorts (HR = 0.46; 95% CI: 0.26–0.81, *P* = 0.0074). **c** Unsupervised clustering of tumors receiving ICI therapy based on the expression pattern of 36 TII-lncRNAs. **d** Correlation between the TISI with the immune score in the Zhao and Miao cohorts. **e** Kaplan–Meier survival curves of tumor patients between TISI_high_ and TISI_low_ groups in the Zhao and Miao cohorts. **f** Association between TISI and ICI responses. Tumors were sorted according to their TISI. **g** ROC curves for ICB response for the TISI, PD-1, PD-L1, and CTLA-4 in two independent immunotherapy cohorts. TISI TIL-lncRNA-derived immune state index, HRs hazard ratios, CI confidence interval, lncRNAs long noncoding RNAs, ICB immune-checkpoint blockade, PD-1 programmed cell death protein 1, PD-L1 programmed death-ligand 1, CTLA-4 cytotoxic T-lymphocyte-associated protein 4, ICIs immune-checkpoint inhibitors, ROC receiver operating characteristic.

By examining the association between the TISI and the expression of ICGs, the TISI was found to be significantly negatively correlated with the expression of 6 ICGs [R = −0.25 and *P* < 0.001 for lymphocyte-activation gene 3, R = −0.15 and *P* < 0.001 for CTLA-4, R = −0.15 and *P* < 0.001 for Fas ligand, R = −0.25 and *P* < 0.001 for T cell immunoreceptor with Ig and ITIM domains, R = −0.14 and *P* < 0.001 for PD-1, and R = −0.29 and *P* < 0.001 for programmed death-ligand 1 (PD-L1); Fig. 5a]. Therefore, it was next investigated whether the TISI had the potential as a genomic tool to predict treatment response to immune-checkpoint inhibitors (ICIs). The prognostic value of the TISI was evaluated by univariate Cox regression analysis within each of the four patient cohorts receiving anti-PD-1 and anti-CTLA-4 treatment, and was integrated using meta-analysis to estimate an overall prognostic effect. The increased TISI was significantly associated with a favorable prognosis following ICI treatment (HR = 0.46, 95% CI = 0.26–0.81, *P* = 0.0074; Fig. 5b). The Zhao and Miao cohorts, which contained detailed clinical information, were further selected as case studies for the validation of the predictive value of the TISI in ICI response. Analysis of the expression pattern of 36 TII-lncRNAs in these two patient cohorts revealed two patient clusters with different immunotherapy responses, which was consistent with the findings of TISI classification (Fig. 5c). Furthermore, the TISI was not only significantly negatively correlated with the immune score of patients (R = −0.48 and *P* = 0.061 for the Miao cohort, and R = −0.54 and *P* < 0.001 for the Zhao cohort; Fig. 5d) but also stratified patients into TISI^high^ and TISI^low^ groups with an apparently different survival (log-rank *P* = 0.038 for the Miao cohort and log-rank *P* = 0.084 for the Zhao cohort; Fig. 5e). The tendency regarding the association between TISI and ICI response indicated that a low TISI revealed significant enrichment in patients that were generally more responsive to ICIs, and a high TISI revealed a predominant enrichment trend in patients that might be more resistant to ICIs (Fig. 5f). Moreover, ROC analysis suggested that the TISI exhibited a predictive superiority or comparable performance (AUC, 0.699 and 0.625) in predicting the response to ICI therapy, as compared with traditional immune biomarkers PD-1, PD-L1, and CTLA-4 (Fig. 5g). These results supported that the TISI is a potential predictive biomarker for the effectiveness of ICI therapy.

## Discussion

The immune system in the TME has been characterized extensively during the past few years, and the understanding of the complexity and dynamics of immunological compositions within the TIME has shed new light on the mechanisms of immune evasion of cancer cells, as well as the discovery of new immunotherapeutic strategies and biomarkers for clinical benefit^30–32^. The establishment and maintenance of specific immunological compositions in the TIME could be affected and regulated by genetic makeup, as well as transcriptional and epigenetic regulators^7,33,34^. Recently, the emerging field of lncRNA-immunity has provided a new perspective on cancer immunity and immunotherapies. Although lncRNAs have been shown to be crucially involved in regulating immune cell functions and diversity within the TIME, lncRNA modifiers of infiltrating immune cells and their contribution to the immunological and clinical phenotype of patients with cancer remain largely uncharacterized.

Given the compelling body of knowledge regarding lncRNAs and tumor-infiltrating immune cells, a systems immunology framework was developed in the present study to identify potential lncRNA modifiers of infiltrating immune cells within the TIME. These modifiers were found to have been strongly correlated with the expression levels of marker genes and infiltrating levels of at least one immune cell type; a significantly differential expression pattern of these modifiers was also observed between tumors with a high and low infiltration for at least one immune cell type. Through integrative analysis of the noncoding transcriptome and immunogenomics profile of 9549 tumor samples across 30 solid cancer types, 36 lncRNAs were identified as modifier candidates underlying immune cell infiltration in the TIME at the pan-cancer level. The results presented herein provided a framework and comprehensive catalog for elucidating the emerging functional roles of lncRNAs in immune modulation.

Previous studies have demonstrated that lncRNA-based molecular subtyping can provide valuable insights into the molecular landscape of cancer, separately from previous protein-coding gene-centered views^35–38^. By focusing on the expression pattern of 36 TIL-lncRNAs, various tumors were subclassified into three pan-cancer subtypes with distinct immunological features, biological behaviors, and disease prognoses. This subtyping scheme expanded the previously existing molecular subtype classification system, and may have clinical implications for TME lncRNA modifiers in terms of prognosis stratification and therapy response prediction. Thus, a TISI index that can predict intratumoral immune and oncogenic states across different cancer types was developed. In addition, the TISI index also had a prognostic value at the pan-cancer level and was able to distinguish between patients with improved and poor survival outcomes. A total of 6 ISs spanning multiple tumor types have previously been reported. Herein, the intersection between the TISI subgroup and ISs was examined, and it was found that TISI_low_ patients tended to be preferentially distributed in the wound healing and IFN-γ dominant subtypes, whereas TISI_high_ patients were preferentially distributed in the inflammatory and immunologically quiet subtypes. For the other two immune types, the TISI was able to stratify patients from the same immune subtype into two risk groups with differential survival time. For transcriptomic molecular subtypes, it was not only shown that the TISI-defined risk groups spanned across CMSs and had no substantial heterogeneity in CMS distribution, but also allowed for a more precise categorization of patients within the same molecular subtype. These findings demonstrated that the TISI stratification scheme is not only primarily driven by the immunological or transcriptomic features alone but also revealed that there is a crosstalk between these features in the TIME.

Immunotherapy has shown great potential as an innovative form of cancer treatment^39^. However, immunotherapeutic responsiveness varies across different cancer types, and even across different patients with the same type of cancer. Emerging evidence has indicated the potential of lncRNAs to predict and guide immunotherapeutic responsiveness^40–42^. Given the association of the TISI with immune and oncogenic states, the clinical relevance of the TISI in cancer immunotherapy was further determined. A sufficient correlation was observed between the TISI and the outcome of and response to cancer immunotherapy in several types of cancer. Furthermore, the TISI revealed a higher predictive performance compared to previous biomarkers (such as PD-1/PD-L1 and CTLA-4) in certain cases, likely due to the additional information on the crosstalk between transcriptomic and immunogenomic features provided by the TISI. These findings further supported the potential of lncRNAs as predictive biomarkers or therapeutic targets in cancer immunotherapy.

In the present study, the impact of lncRNA expression on immune-related molecular and cellular components in the TIME at the pan-cancer level was systematically investigated, and lncRNA modifier candidates of infiltrating immune cells within the TIME were identified, providing a comprehensive resource and view for future functional and mechanistic investigations of lncRNA-mediated cancer immunity. In addition, the clinical relevance of these TIL-lncRNAs with survival and response to cancer immunotherapy presented herein highlighted the future potential of the clinical application of lncRNA-based immunotherapeutic strategies in precision immunotherapy.

## Methods

### Pan-cancer patient and immunotherapy data

The Cancer Genome Atlas (TCGA) multi-omics data were obtained from UCSC Xena (https://xenabrowser.net/datapages/)^43^, including RNA-seq with HiSeq Illumina platform transformed by log(x + 1), copy number variation with the Illumina platform estimated using the GISTIC2 method, and somatic mutation with the Illumina platform. A total of 9549 tumor samples across 30 solid cancer types were analyzed in this study.

Transcriptomic data (HiSeq Illumina platform) and clinical information from patients with tumors treated with programmed cell death protein 1 (PD-1)/cytotoxic T-lymphocyte-associated protein 4 (CTLA-4) blockade therapy were obtained from previously published prospective clinical trials, including from 16 patients with clear metastatic cell renal cell carcinoma (ccRCC) from Miao’s study (referred to as the Miao cohort)^44^, 34 patients with glioblastoma multiforme (GBM) from Zhao’s study (referred to as the Zhao cohort)^45^, 37 patients with metastatic melanoma from VanAllen’s study (referred to as the VanAllen cohort)^46^, and 76 patients with melanoma from Gide’s study (referred to as the Gide cohort)^47^. Ethical approval was not needed because these datasets are allowed to be publicly available.

### Transcriptome deconvolution of the TIME

The abundance of infiltrating immune cell populations in the TIME was estimated by deconvolution methods using the CIBERSORT with LM22 signature matrix^48^. The fraction of stromal and immune cells in the tumor samples was calculated by the single-sample geneset enrichment analysis (ssGSEA) using ESTIMATE^49^.

### Identification of lncRNA modifiers of tumor-infiltrating immune cells

lncRNA expression profiles of TCGA pan-cancer cases were obtained based on the lncRNA annotation of the GENCODE project^50^. Following the removal of the lncRNAs with no expression in >20% of total patients^51^, a total of 4755 lncRNAs were kept for further analysis. lncRNA modifiers of tumor-infiltrating immune cells (TII-lncRNAs) were identified using a systems immunology framework as follows (Fig. 1a): (i) All lncRNAs were ranked based on their co-expression relationship with immune marker genes, and lncRNAs with a significantly higher Pearson correlation coefficient (PCC) of 0.3 and *P* < 0.05 were considered as immune gene-related lncRNAs; (ii) these immune gene-related lncRNAs were ranked based on the correlation between their expression and the abundance of a given infiltrating immune cell population, as calculated by PCC, and those with a significantly higher PCC were considered as candidate TII-lncRNAs; (iii) samples were classified into high and low immune infiltration groups using the top and bottom quartiles for a given immune cell population. Candidate TII-lncRNAs that were significantly differentially expressed between tumors with a high and low immune infiltration were defined as TII-lncRNAs.

### Computational index of cancer-related events and immunomodulation

To quantify the role and dynamics of epithelial-mesenchymal transition (EMT) in each patient, 94 mesenchymal and 111 epithelial cell marker genes were obtained from a previous study^52^, and the EMT score was calculated using the Student’s *t*-test score between epithelial cell marker genes and the expression of mesenchymal cell marker genes^53^. To characterize the occurrence of immune-mediated tissue-specific destruction, the Immunologic Constant of Rejection (ICR) score was calculated using the mean of the normalized log2 transformed expression values of 20 ICR signature genes from Roeland’s study^54^. To quantify the immune effector activity in solid tumors, the cytolytic activity (CYT) score was calculated using the geometric mean of two key cytolytic effectors, granzyme A, and perforin, in each patient^55^.

### Development of TII-lncRNA-derived immune state index (TISI)

The TISI was calculated using the mean expression levels of 36 TII-lncRNAs. Samples with a higher TISI exhibited a low-affinity immune phenotype, whereas a lower TISI reflected a high-affinity immune phenotype.

### Enrichment analysis of functional genesets

Single-sample Gene Set Enrichment Analysis (ssGSEA) was performed to calculate the enrichment score (ES) of each patient using R package ‘GSVA’^56^ and identify up- or downregulated interested genesets or pathways in different subtypes within each tumor type. The immunologic signature and hallmark genesets were obtained from the Molecular Signatures Database (MSigDB, V7.2)^57^.

### Statistical analysis

Consensus clustering analysis was performed on the R package ‘ConsensusClusterPlus’ using the K-means method and Euclidean distances to identify the optimum number of clusters in pan-cancer based on the expression pattern of TII-lncRNAs. Univariate and multivariate Cox proportional hazards regression models were used to assess the association between the TISI and overall survival with/without clinical variables. The hazard ratio (HR) and 95% confidence interval (CI) were calculated. Two-sided Wilcoxon rank-sum tests were used to compare two groups. The Kaplan–Meier method and log-rank test were conducted to compare survival differences between two tumor groups. Receiver operating characteristic (ROC) curves were used to evaluate the predictive performance for the response to immunotherapy, and the area under the curve (AUC) was calculated. All statistical analysis was performed using R/Bioconductor (version 3.6.1).

### Reporting summary

Further information on research design is available in the Nature Research Reporting Summary linked to this article.

## Supplementary information

Supplementary Information

Reporting Summary

## Data Availability

The Cancer Genome Atlas (TCGA) pan-cancer data were obtained from UCSC Xena (https://gdc-hub.s3.us-east-1.amazonaws.com/download/GDC-PANCAN.htseq_fpkm-uq.tsv.gz), including adrenocortical carcinoma (ACC, *n* = 78), bladder urothelial carcinoma (BLCA, *n* = 399), breast invasive carcinoma (BRCA, *n* = 1066), cervical squamous cell carcinoma and endocervical adenocarcinoma (CESC, *n* = 284), cholangiocarcinoma (CHOL, *n* = 35), colon adenocarcinoma (COAD, *n* = 431), esophageal carcinoma (ESCA, *n* = 151), glioblastoma multiforme (GBM, *n* = 154), head and neck squamous cell carcinoma (HNSC, *n* = 495), kidney chromophobe (KICH, *n* = 63), kidney renal clear cell carcinoma (KIRC, *n* = 522), kidney renal papillary cell carcinoma (KIRP, *n* = 284), brain lower grade glioma (LGG, *n* = 508), liver hepatocellular carcinoma (LIHC, *n* = 364), lung adenocarcinoma (LUAD, *n* = 498), lung squamous cell carcinoma (LUSC, *n* = 489), mesothelioma (MESO, *n* = 78), ovarian serous cystadenocarcinoma (OV, *n* = 358), pancreatic adenocarcinoma (PAAD, *n* = 176), prostate adenocarcinoma (PRAD, *n* = 481), rectum adenocarcinoma (READ, *n* = 154), sarcoma (SARC, *n* = 259), skin cutaneous melanoma (SKCM, *n* = 454), stomach adenocarcinoma (STAD, *n* = 348), testicular germ cell tumors (TGCT, *n* = 137), thyroid carcinoma (THCA, *n* = 503), thymoma (THYM, *n* = 117), uterine corpus endometrial carcinoma (UCEC, *n* = 534), uterine carcinosarcoma (UCS, *n* = 53), uveal melanoma (UVM, *n* = 76). Transcriptomic data (HiSeq Illumina platform) and clinical information of four immunotherapy datasets were obtained from previously published prospective clinical trials, including Miao’s study^44^, Zhao’s study^45^, VanAllen’s study^46^, and Gide’s study^47^.
