# Supplementary material for: Pan-cancer characterization of lncRNA modifiers of immune microenvironment reveals clinically distinct de novo tumor subtypes
Source: NPJ Genom Med. 2021 Jun 17;6:52. doi: 10.1038/s41525-021-00215-7 (PMC8211863; doi:10.1038/s41525-021-00215-7)
Supplement: Supplementary file 1 — Supplementary Information [file 41525_2021_215_MOESM1_ESM.pdf]

Supplementary Information

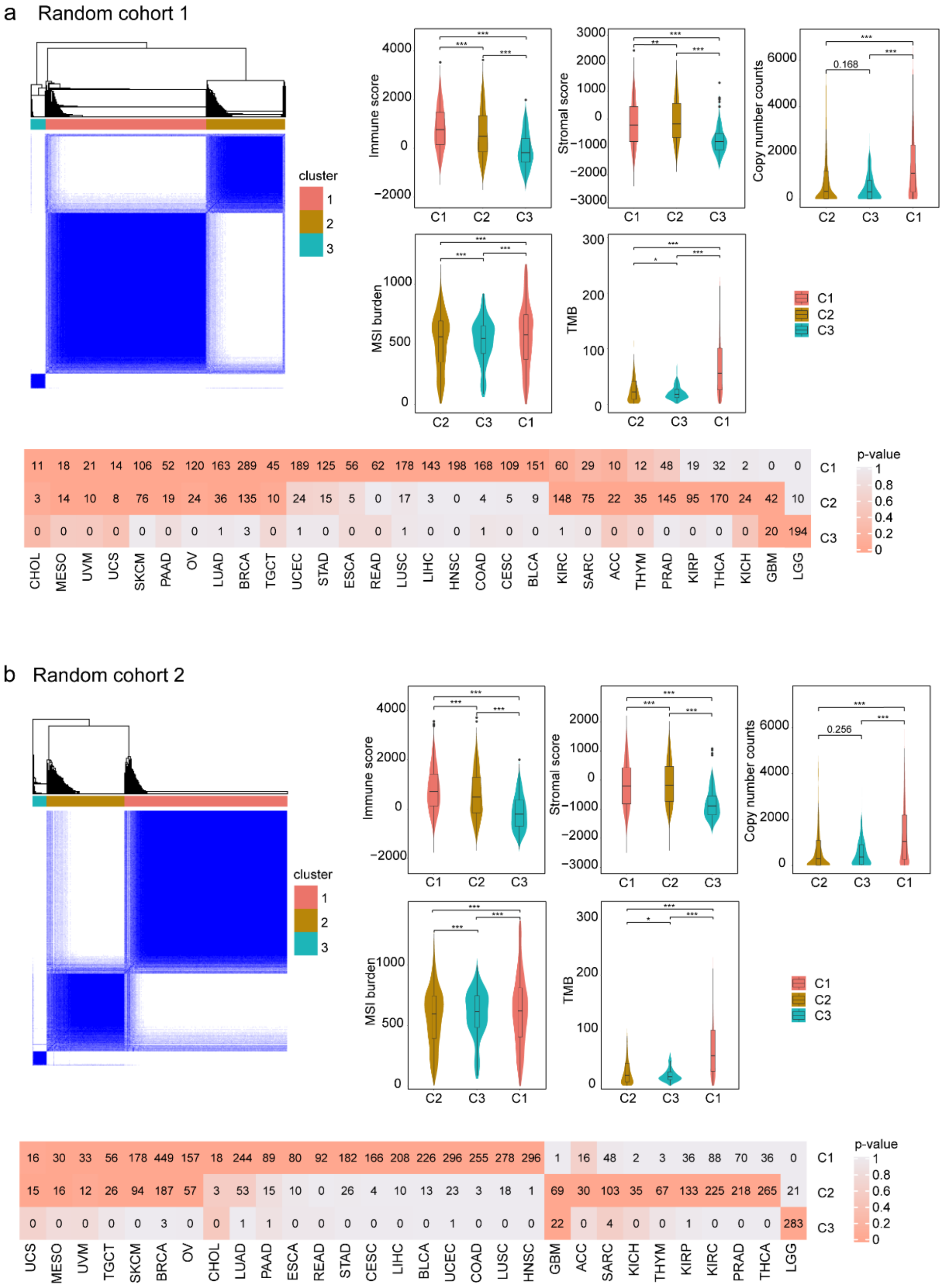

Supplementary Figure 1. The exploration and verification of TII-lncRNAs.

Consensus clustering was performed to divided the training dataset (A) and internal

validation dataset (B) into three cluster; Distribution of immunology and epigenetics among different clusters using boxplot; Number of cases in each cluster across tumor types. Colors in boxes were used to represent the p-value calculated from a hypergeometric test which comparing the proportion of samples with a known cancer type in a cluster to the proportion of samples which were in that cluster overall. The level of enrichment was indicated by red color. *** p<0.001, **p<0.01 and * p<0.05; P-values for two-sided Wilcoxon's rank-sum tests.

Supplementary Table 1. List of TIME lncRNA modifiers

ENSG_name	Gene_symbol
ENSG00000215067	ALOX12-AS1
ENSG00000253982	AC100810.1
ENSG00000223891	OSER1-DT
ENSG00000225791	TRAM2-AS1
ENSG00000255455	AP003486.1
ENSG00000248508	SRP14-AS1
ENSG00000266904	LINC00663
ENSG00000224281	SLC25A5-AS1
ENSG00000280047	AC091825.3
ENSG00000227398	KIF9-AS1
ENSG00000248092	NNT-AS1
ENSG00000279453	Z99129.4
ENSG00000258057	BCDIN3D-AS1
ENSG00000274220	AC009163.7
ENSG00000260917	AL158212.3
ENSG00000258824	AL122035.1
ENSG00000230319	TTLL1-AS1
ENSG00000260464	AL049796.1
ENSG00000267049	AC002398.1
ENSG00000279722	AC007342.7
ENSG00000234456	MAGI2-AS3
ENSG00000248668	OXCT1-AS1
ENSG00000271265	AL355297.2
ENSG00000271743	AF287957.1
ENSG00000246308	AC116535.2

ENSG00000255389	Z97989.1
ENSG00000259969	AL049838.1
ENSG00000273419	AC004877.1
ENSG00000267532	MIR497HG
ENSG00000225706	PTPRD-AS1
ENSG00000243069	ARHGEF26-AS1
ENSG00000260118	AL157700.1
ENSG00000280255	AC004947.2
ENSG00000225938	AL109741.1
ENSG00000277351	AC013553.3
ENSG00000226252	AL135960.1
