# Supplementary material for: Pan-cancer characterization of lncRNA modifiers of immune microenvironment reveals clinically distinct de novo tumor subtypes
Source: NPJ Genom Med. 2021 Jun 17;6:52. doi: 10.1038/s41525-021-00215-7 (PMC8211863; doi:10.1038/s41525-021-00215-7)
Supplement: Supplementary file 2 — Reporting Summary [file 41525_2021_215_MOESM2_ESM.pdf]

nature research

Corresponding author(s): Meng Zhou, Jie Sun

Last updated by author(s): May 24, 2021

Reporting Summary

Nature Research wishes to improve the reproducibility of the work that we publish. This form provides structure for consistency and transparency in reporting. For further information on Nature Research policies, see our Editorial Policies and the Editorial Policy Checklist.

Statistics

For all statistical analyses, confirm that the following items are present in the figure legend, table legend, main text, or Methods section.

n/a	Confirmed	
☐	☒	The exact sample size (n) for each experimental group/condition, given as a discrete number and unit of measurement
☒	☐	A statement on whether measurements were taken from distinct samples or whether the same sample was measured repeatedly
☐	☒	The statistical test(s) used AND whether they are one- or two-sided<br>*Only common tests should be described solely by name; describe more complex techniques in the Methods section.*
☐	☒	A description of all covariates tested
☐	☒	A description of any assumptions or corrections, such as tests of normality and adjustment for multiple comparisons
☐	☒	A full description of the statistical parameters including central tendency (e.g. means) or other basic estimates (e.g. regression coefficient) AND variation (e.g. standard deviation) or associated estimates of uncertainty (e.g. confidence intervals)
☐	☒	For null hypothesis testing, the test statistic (e.g. F, t, r) with confidence intervals, effect sizes, degrees of freedom and P value noted<br>*Give P values as exact values whenever suitable.*
☒	☐	For Bayesian analysis, information on the choice of priors and Markov chain Monte Carlo settings
☒	☐	For hierarchical and complex designs, identification of the appropriate level for tests and full reporting of outcomes
☐	☒	Estimates of effect sizes (e.g. Cohen's d, Pearson's r), indicating how they were calculated

Our web collection on statistics for biologists contains articles on many of the points above.

Software and code

Policy information about availability of computer code

Data collection	no software was used to data collection
Data analysis	no software was used

For manuscripts utilizing custom algorithms or software that are central to the research but not yet described in published literature, software must be made available to editors and reviewers. We strongly encourage code deposition in a community repository (e.g. GitHub). See the Nature Research guidelines for submitting code & software for further information.

Data

Policy information about availability of data

All manuscripts must include a data availability statement. This statement should provide the following information, where applicable:

- Accession codes, unique identifiers, or web links for publicly available datasets
- A list of figures that have associated raw data
- A description of any restrictions on data availability

The Cancer Genome Atlas (TCGA) pan-cancer data were obtained from UCSC Xena(https://gdc-hub.s3.us-east-1.amazonaws.com/download/GDC-PANCAN.htseq_fpkm-uq.tsv.gz), including adrenocortical carcinoma (ACC, n=78), bladder urothelial carcinoma (BLCA, n = 399), breast invasive carcinoma (BRCA, n =1066), cervical squamous cell carcinoma and endocervical adenocarcinoma (CESC, n=284), cholangiocarcinoma (CHOL, n=35), colon adenocarcinoma (COAD, n=431), esophageal carcinoma (ESCA, n=151), glioblastoma multiforme (GBM, n=154), head and neck squamous cell carcinoma (HNSC, n=495), kidney chromophobe (KICH, n=63), kidney renal clear cell carcinoma (KIRC, n=522), kidney renal papillary cell carcinoma (KIRP, n=284), brain lower grade glioma (LGG, n=508), liver hepatocellular carcinoma (LIHC, n=364), lung adenocarcinoma (LUAD, n=498), lung squamous cell carcinoma (LUSC, n=489), mesothelioma (MESO, n=78), ovarian serous cystadenocarcinoma (OV, n=358), pancreatic adenocarcinoma (PAAD, n=176), prostate adenocarcinoma (PRAD, n=481), rectum adenocarcinoma (READ, n=154), sarcoma (SARC, n=259), skin cutaneous melanoma (SKCM, n=454), stomach adenocarcinoma (STAD, n=348), testicular germ cell

tumors (TGCT, n=137), thyroid carcinoma (THCA, n=503), thymoma (THYM, n=117), uterine corpus endometrial carcinoma (UCEC, n=534), uterine carcinosarcoma (UCS, n=53), uveal melanoma (UVM, n=76).
Transcriptomic data (HiSeq Illumina platform) and clinical information of four immunotherapy datasets were obtained from previously published prospective clinical trials, including Miao's study43, Zhao's study44, VanAllen's study 45 and Gide's study 46.

Field-specific reporting

Please select the one below that is the best fit for your research. If you are not sure, read the appropriate sections before making your selection.

☒ Life sciences ☐ Behavioural & social sciences ☐ Ecological, evolutionary & environmental sciences

For a reference copy of the document with all sections, see nature.com/documents/nr-reporting-summary-flat.pdf

Life sciences study design

All studies must disclose on these points even when the disclosure is negative.

Sample size	A total of 9,549 tumor samples across 30 solid cancer types were analyzed in this study.
Data exclusions	so
Replication	comfirm
Randomization	A total of 9,549 tumor samples across 30 solid cancer types were obtained from The Cancer Genome Atlas (TCGA)
Blinding	A total of 9,549 tumor samples across 30 solid cancer types were obtained from The Cancer Genome Atlas (TCGA)

Reporting for specific materials, systems and methods

We require information from authors about some types of materials, experimental systems and methods used in many studies. Here, indicate whether each material, system or method listed is relevant to your study. If you are not sure if a list item applies to your research, read the appropriate section before selecting a response.

Materials & experimental systems

n/a	Involved in the study	
☒	☐	Antibodies
☒	☐	Eukaryotic cell lines
☒	☐	Palaeontology and archaeology
☒	☐	Animals and other organisms
☒	☐	Human research participants
☒	☐	Clinical data
☒	☐	Dual use research of concern

Methods

n/a	Involved in the study	
☒	☐	ChIP-seq
☒	☐	Flow cytometry
☒	☐	MRI-based neuroimaging
